# Disease Burden of RSV Infections and Bronchiolitis in Young Children (< 5 Years) in Primary Care and Emergency Departments: A Systematic Literature Review

**DOI:** 10.1111/irv.13344

**Published:** 2024-08-04

**Authors:** Susanne Heemskerk, Lotte van Heuvel, Tamana Asey, Mathieu Bangert, Rolf Kramer, John Paget, Jojanneke van Summeren

**Affiliations:** ^1^ Netherlands Institute for Health Services Research (Nivel) Utrecht The Netherlands; ^2^ Ecole des Hautes Etudes en Santé Publique (EHESP) Rennes France; ^3^ Sanofi Vaccines Lyon France

**Keywords:** child, emergency department, Global Burden of Disease, primary healthcare, respiratory syncytial virus

## Abstract

Respiratory syncytial virus (RSV) is the most common cause of acute respiratory infections in young children. Limited data are available on RSV disease burden in primary care and emergency departments (EDs). This review synthesizes the evidence on population‐based incidence rates of RSV infections in young children (< 5 years) in primary care and EDs. A systematic literature review was performed in PubMed and Embase. Studies reporting yearly population‐based RSV incidence rates in primary care and EDs were included. A total of 4244 records were screened and 32 studies were included, conducted between 1993 and 2019. Studies were mainly performed in high‐income countries (*n* = 27), with 15 studies in North America and 10 studies in Europe. There was significant variability in study methodology and setting among studies, resulting in considerable variability in reported incidence rates. Incidence rates were higher in primary care—ranging from 0.8 to 330 (median = 109) per 1000 population—compared to EDs (7.5–144.0, median = 48). The highest incidence rates were reported in infants. Additionally, incidence rates were higher in high‐income countries and in studies using laboratory‐confirmed RSV cases compared to studies using bronchiolitis ICD‐codes (non–laboratory confirmed). Our study found that a substantial number of children under 5 years of age attend primary care settings and EDs, every year for RSV infections. Due to the considerable heterogeneity in study methodology, it was impossible to draw definitive conclusions regarding factors explaining differences in reported incidence rates. Additionally, more studies in low‐ and middle‐income countries are recommended.

## 1 Introduction

Respiratory infections are one of the leading causes of morbidity and mortality among young children worldwide [1]. Respiratory syncytial virus (RSV) is considered the most common pathogen causing respiratory infections in young children [2, 3]. In a recent birth cohort study from Western Europe, 1 in every 56 healthy term‐born infants was hospitalized for an RSV infection [4]. Hospitalizations and deaths due to RSV are greatest in infants younger than 6 months [2, 3], with the majority of deaths occurring in countries with low income levels [2].

Even though the global burden of disease of RSV in the community and hospital setting has been extensively studied [3, 5], the burden of disease in primary care remains unclear. According to the disease burden pyramid [6], the incidence of RSV infections is expected to be highest in the community setting (which includes mild and asymptomatic infections that do not require medical attention) and gradually decreases in primary care and hospital settings (including more severe infections). It is crucial to assess the burden of disease on the whole health system, including primary care, to make informed decisions on prevention strategies.

Treatment of RSV infections is limited to supportive care; however, there are several promising new developments in the field of preventive strategies [7]. Until 2022, the monoclonal antibody palivizumab was the only preventive measure used in practice, but it was limited to high‐risk infants, due to the need for monthly injections, limited effectiveness, and high cost [8, 9]. As of 2022, a new long‐acting monoclonal antibody (nirsevimab) and maternal vaccine have been market approved in Europe and the United States, and other monoclonal antibodies and vaccine candidates are in late stages of clinical development [10, 11].

Understanding the RSV disease burden is crucial for informed decision‐making regarding the potential introduction of these future prophylactic strategies. This includes the population‐based RSV incidence rates in primary care and emergency departments (EDs). Our systematic literature review is therefore aimed at broadly synthesizing the population‐based disease burden of RSV in children < 5 years in primary care and EDs.

## 2 Methods

### 2.1 Study Selection

A systematic literature search was performed in PubMed and Embase and updated on 7 November 2022. The database search was performed using terms related to pathogen and disease (#1) and setting and healthcare provider (#2) (Supporting Information S1: Supplemental Digital Content 1). No language restrictions were applied. Only published, full‐text research articles were assessed.

The population of interest comprised children younger than 5 years of age with an RSV infection in primary care settings—general practitioners (GPs), pediatric offices (POs), and outpatient departments (OPDs)—and EDs. Studies reporting the yearly or seasonal population‐based incidence rate of RSV (including bronchiolitis as proxy for RSV), from now on referred to as yearly incidence rates, were eligible for inclusion. Both observational and modelling studies were included. Exclusion criteria were studies published before December 1999, studies reporting only the proportion of RSV‐positive cases among the sampled specimens (no population‐based numbers), and the following article types: review articles, conference abstracts, case reports, and commentary articles.

All titles and abstracts were independently screened for inclusion by two researchers (TA or LvH or SH). Conflicts were discussed with a third researcher (JvS or MB or RK) until consensus was reached. The full texts of studies eligible for inclusion were retrieved and read by two researchers independently (LvH and SH).

### 2.2 Data Extraction

Two researchers (LvH and SH) independently extracted data from the eligible papers; a third reviewer (JvS) was approached for resolution when the reviewers did not agree. Information extracted included among others: study method, laboratory confirmation, case definition for sampling, age group, country, and setting. Study authors were contacted when data on incidence rates were missing.

### 2.3 Critical Appraisal

The included studies were critically appraised using the Joanna Briggs Institute critical appraisal checklist for prevalence studies (Supporting Information S1: Supplemental Digital Content 2) [12]. The purpose of this appraisal is to identify potential biases in design, conduct, and analysis for the calculation of population‐based incidence rates of RSV infections. Two researchers (LvH and SH) independently assessed each item as having a risk of bias, no risk of bias, or unclear risk of bias. Discrepancies were resolved by discussing them with a third researcher (JvS). Since the checklist was not perfectly suitable for the purpose of this review, the questions regarding the sample size adequacy, sample coverage, and response rate were not assessed.

### 2.4 Data Synthesis and Analysis

All reported incidence rates were transformed into incidence rates per 1000 population per year. If studies reported multiple RSV‐associated incidence rates for several case definitions such as RSV‐associated lower respiratory tract infection (LRTI) and bronchiolitis, the incidence rates using the most narrow (RSV) case definition (in this case, bronchiolitis) were chosen and reported. The age groups were categorized as < 6 months, < 1 year, < 2 years, and < 5 years. For certain studies, we used age‐specific national population estimates to calculate the pooled incidence rate over multiple years and the incidence rates for missing age categories. Country income level classification was derived from the World Bank [13].

The heterogeneity in study methodology and key study characteristics was evaluated and analyzed to elucidate the variation in incidence rates. These key characteristics included care setting, age, case definition, country income level, world region, seasonality, and subnational versus national data. The data were analyzed using R version 5.0 (R Core Team, 2020) and RStudio (Rstudio Team, 2020).

## 3 Results

### 3.1 Study Selection

**FIGURE 1:**
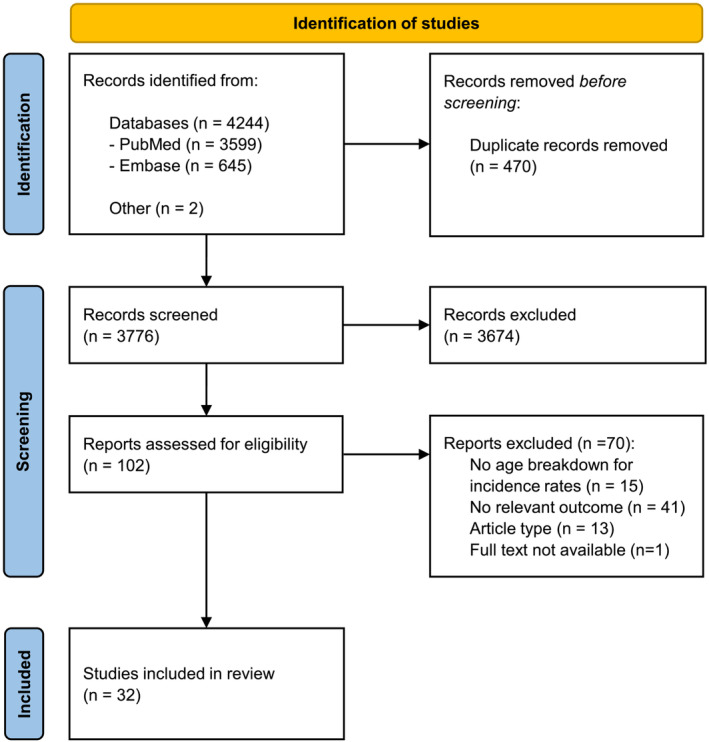
PRISMA flow diagram of the study screening and selection process.

A total of 4244 records were retrieved in the literature search, and two additional records were identified through an expert (Figure 1). An overview of study characteristics of the 32 included studies is provided in Table 1 and Supporting Information S2: Supplemental Digital Content 4 [14, 15, 16, 17, 18, 19, 20, 21, 22, 23, 24, 25, 26, 27, 28, 29, 30, 31, 32, 33, 34, 35, 36, 37, 38, 39, 40, 41, 42, 43, 44, 45].

**TABLE 1:** Study characteristics of 32 included studies.

Author	Year Data^a^	Country^b^	Income level	Study methods^c^	Case definition for sampling^d^	Laboratory confirmed^e^	Setting	Age group	Yearly RSV incidence rate (per 1000 population)
Ambrose et al. [14]	2009–2011 (s)	USA (n)	HIC	Cohort	ARI	Yes	OPD (LRI)	0–6 months	330 (279–388)
							ED	0–6 months	141 (108–179)
Barbieri et al. [15]	2012–2019 (y)	Italy (n)	HIC	Database	Bronchiolitis	No	PO	0–5 months	111.9 (108.3–115.6)
								0–11 months	89.9 (87.7–92.0)
								0–23 months	46.6 (45.5–47.6)
Bourgeois et al. [16]	2003–2005 (s)	USA (n)	HIC	Cohort	ARI	Yes	ED	0–23 months	64.4 (45.4–91.3)
Bourgeois et al. [17]	1993–2004 (s)	USA (s)	HIC	Surveillance	ARI	Yes	ED	0–5 months	144.0 (140–148)
								0–23 months	75.7 (73.8–77.7)
								0–59 months	40.4 (39.3–41.5)
Brunet et al. [18]	2004–2018 (y)	Canada (s)	HIC	Database	Bronchiolitis	No	ED	0–23 months	29.1 (28.9–29.3)
Bueno Campaña et al. [19]	2003 (s)	Spain (s)	HIC	Cohort	ARI	Yes	PO	0–5 months	119
Cromer et al. [20]	2017 (y)	England (n)	HIC	Modelling (s)	ARI	Yes	GP	0–5 months	214.2 (211.3–217)
								0–59 months	119.9 (118.6–121.2)
Dolk et al. [21]	2003–2014 (s)	Netherlands (n)	HIC	Surveillance	ILI	Yes	GP	0–59 months	17.6
Forster et al. [22]	1999–2001 (y)	Germany (n)	HIC	Cohort	LRTI	Yes	PO	0–35 months	77 (67–89)
Hall et al. [23]	2002–2004 (s)	USA (s)	HIC	Surveillance	ARI	Yes	PO	0–11 months	155 (54–447)
								0–23 months	110 (36–346)
								0–59 months	80 (36–179)
							ED	0–11 months	56 (22–144)
								0–23 months	44 (17–118)
								0–59 months	28 (15–50)
Hasegawa et al. [24]	2006 and 2010 (y)	USA (n)	HIC	Database	Bronchiolitis	No	ED	0–11 months	53.9
								0–23 months	35.9
Heikkinen, Ojala, and Waris [25]	2000–2002 (s)	Finland (s)	HIC	Cohort	Pneumonia	Yes	OPD	0–11 months	263 (133–479)
								0–23 months	263 (153–431)
								0–59 months	212 (133–327)
Jackson et al. [26]	2018–2019 (s)	USA (s)	HIC	Surveillance	ARI	Yes	OPD	12–23 months	107
								24–59 months	± 60
Kamigaki et al. [27]	2012–2014 (y)	Philippines (s)	LMIC	Surveillance	ILI	Yes	OPD	0–5 months	7.2 (3.0–14.7)
								0–23 months	19.0 (11.7–29.5)
								0–59 months	13.0 (7.1–22.0)
Law et al. [28]	2001–2003 (s)	Canada (n)	HIC	Cohort	RTI	Yes	ED	0–6 months	82.7
Lively et al. [29]	2004–2009 (s)	USA (s)	HIC	Surveillance	ARI	Yes	PO	0–5 months	215.7 (179.8–251.5)
								0–11 months	230.9 (192.5–269.3)
								0–23 months	205.7 (169.5–241.9)
							ED	0–5 months	74.8 (64.0–85.6)
								0–11 months	66.2 (56.6–75.7)
								0–23 months	59.6 (50.9–68.3)
Mansbach, Emond, and Camargo [30]	1992–2000 (y)	USA (n)	HIC	Database	Bronchiolitis	No	ED	0–23 months	26 (22–31)
Mansbach, Pelletier, and Camargo [31]	1993–2004 (y)	USA (n)	HIC	Database	Bronchiolitis	No	OPD	0–23 months	103 (83–124)
Marcone et al. [32]	2008–2010 (y)	Argentina (s)	UMIC	Cohort	ARI	Yes	ED	0–71 months	352 (167–557)
Moore et al. [33]	2001–2005 (y)	Australia (s)	HIC	Database	Bronchiolitis	No	ED	0–11 months	51.0
								0–23 months	29.6
								0–59 months	11.8
Muñoz‐Quiles et al. [34]	2009–2012 (y)	Spain (s)	HIC	Database	Bronchiolitis	No	PO	0–5 months	190
								0–11 months	197
								0–23 months	143
Okiro et al. [35]	2002–2004 (y)	Kenya (s)	LMIC	Surveillance	ARI	Yes	OPD	0–11 months	14.2 (11.9–17.1)
								0–59 months	7.7 (6.5–9)
Paget et al. [36]	2002–2008 (s)	England (n)	HIC	Modelling (s)	ILI	Yes	GP	0–59 months	0.8
Paget et al. [36]	2002–2008 (s)	Netherlands (n)	HIC	Modelling (s)	ILI	Yes	GP	0–59 months	3.8
Prasad et al. [37]	2014–2016 (s)	New Zealand (s)	HIC	Surveillance	ARI	Yes	ED	0–11 months	34.4 (32.1–36.7)
Rainisch et al. [38]	2002–2009 (y)	USA (n)	HIC	Modelling (s)	LRTI	Yes	OPD	0–11 months	230.9 (71.0–337.2)
							ED	0–11 months	66.2 (16.8–132.7)
Reyes Domínguez et al. [39]	2016–2019 (y)	Spain (s)	HIC	Database	RSV	Yes	ED	0–24 months	53.4
Rosychuk et al. [40]	1999–2005 (y)	Canada (s)	HIC	Database	Bronchiolitis	No	ED	0–35 months	38.2
Rowlinson et al. [41]	2011–2012 (y)	Egypt (s)	LMIC	Surveillance	ILI	Yes	OPD	12–59 months	23 (18.1–28)
Suh et al. [42]	2011–2019 (y)	USA (n)	HIC	Database	RSV	No	ED	0–11 months	33.8 (31.7–35.9)
Tempia et al. [43]	2013–2015 (y)	South Africa (s)	UMIC	Surveillance	ILI	Yes	OPD	0–11 months	79.8 (53.6–111.4)
								0–59 months	47.7 (33.8–65.8)
Thomas et al. [44]	2017–2018 (s)	Finland (s)	HIC	Cohort	RTI	Yes	OPD	0–3 months	328.4 (275.2–389.0)
To et al. [45]	2008–2019 (y)	Canada (s)	HIC	Database	Bronchiolitis	No	ED	0–11 months	20.7
								0–59 months	7.5

Abbreviations: ARI, acute respiratory infection; ED, emergency department; GP, general practitioner; HIC, high‐income country; ILI, influenza‐like illness; LMIC, lower‐middle‐income country; LRTI, lower respiratory tract infection; OPD, outpatient department; PO, pediatric office; RSV, respiratory syncytial virus; RTI, respiratory tract infection; UMIC, upper‐middle‐income country.

^a^ y, year‐round data; s, seasonal data.

^b^ n, national data; s, subnational data.

^c^ s, surveillance data.

^d^ The case definition describes the population eligible for RSV testing. The incidence rates reported are RSV‐related, for example, RSV‐associated ARI rates.

^e^ For studies that used surveillance data (see study methods): not all cases are laboratory confirmed, instead RSV incidence rates are calculated based on, for example, ARI incidence rates adjusted for the proportion RSV‐positive cases.

### 3.2 Description of Study Characteristics

Out of 32 studies, 19 studies reported RSV incidence rates in primary care settings: OPD (*n* = 10), PO (*n* = 6), and GP (*n* = 3). RSV incidence rates in EDs were reported in 17 studies, with 4 reporting rates in both primary care and EDs. Only 3 were modelling‐based studies.

Different case definitions for sampling were identified: acute respiratory infection (ARI) (*n* = 11), influenza‐like illness (ILI) (*n* = 5), (L)RTI (*n* = 4), pneumonia (*n* = 1), bronchiolitis (*n* = 9), and unspecified RSV (*n* = 2). RSV was laboratory confirmed in most studies (*n* = 22). Among the 10 non–laboratory‐confirmed studies, ICD‐9 and ICD‐10 codes specific for RSV or bronchiolitis were used for case identification.

**FIGURE 2:**
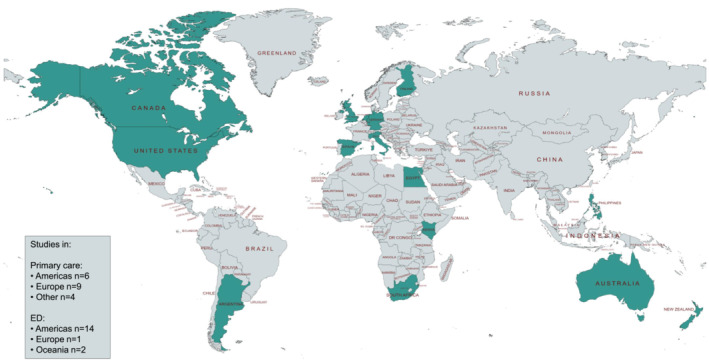
World map showing the location of 32 included studies reporting population‐based incidence rates of RSV in primary care and emergency departments (EDs) in children < 5 years. Four studies reported incidence rates in both primary care and EDs. Studies were conducted in the Americas (*n* = 16), Europe (*n* = 10), Oceania (*n* = 2), Africa (*n* = 2), Asia (*n* = 1), and Middle East (*n* = 1). RSV indicates respiratory syncytial virus.

The location of the included studies is shown in Figure 2 and Table 1. All data were collected between 1993 and 2019 (pre‐COVID‐19), with most studies (*n* = 27) reporting incidence rates over multiple years.

### 3.3 Critical Appraisal of Included Studies

The quality of the studies estimating population‐based RSV incidence rates was appraised as their ability to find valid and unbiased estimates (Supporting Information S1: Supplemental Digital Content 3). Overall, we have rated the quality of the studies as moderate. For the criteria “description of study subjects and setting” (Criterion 3) and “measurement of the condition” (Criterion 5), all studies scored no risk of bias. The majority of the studies (*n* = 30) used valid methods, such as laboratory confirmation or ICD‐codes, for the identification of RSV (Criterion 4). In two studies, specific testing methods for RSV were unclear. Furthermore, the sample frame was appropriate in 21 studies (Criterion 1). Among the 11 studies with risk of bias due to an inappropriate sample frame, there were 2 that included only at‐risk patients and 9 that reported only bronchiolitis incidence rates as a proxy for RSV. In 21 studies, the statistical analysis was scored as no risk of bias; among the other 11 studies, a 95% confidence interval (CI) was not presented (Criterion 6). Lastly, 18 studies used appropriate recruitment methods (e.g., random sample), while 12 lacked a clear description of recruitment and 2 did not recruit participants appropriately (criterion 2).

### 3.4 Population‐Based Incidence Rates of RSV

**FIGURE 3:**
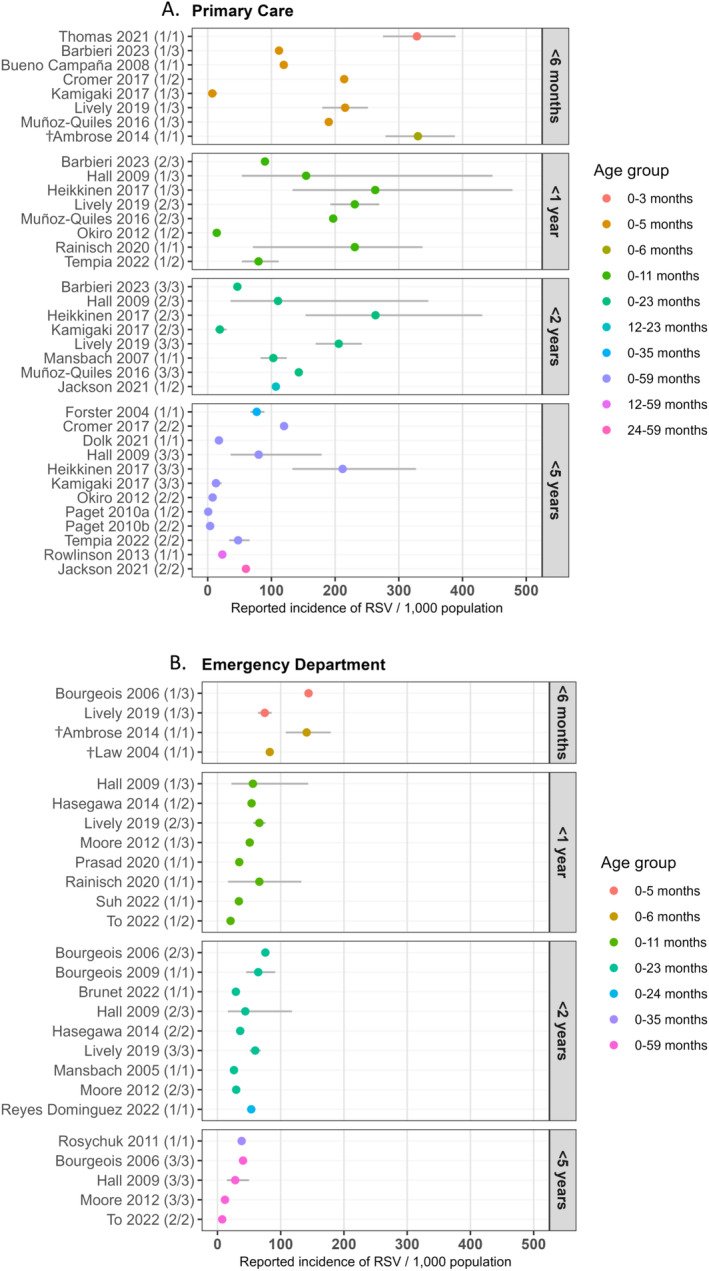
Population‐based yearly incidence rate per 1000 population and 95% CI of RSV infection in children by age category in (A) primary care and (B) emergency departments. Some studies reported incidence rates in multiple age categories; the first of two age categories is reported as (1/2), etc. †Only including at‐risk preterm infants born at 32–35 weeks gestational age not receiving RSV prophylaxis. RSV indicates respiratory syncytial virus.

The population‐based RSV incidence rates in primary care (Figure 3A) and EDs (Figure 3B) specified by age category are shown in Figure 3. The impact of four key study characteristics (care setting, age, case definition, and country income level) on the reported incidence rates is described below.

#### 3.4.1 Setting

In primary care, the yearly incidence rates in all age categories varied widely, ranging from 0.8 to 330 (median = 109) per 1000 population. In EDs, the reported incidence rates were generally lower compared to primary care and showed more uniformity. These incidence rates ranged from 7.5 to 144.0 (median = 48) per 1000 population per year in all age categories. The incidence rate reported by Marcrone et al. (2015) [32]—352 (95% CI, 167–557) per 1000 population per year—was substantially higher compared to the other ED rates and was considered an outlier and is therefore not shown in the figures or used for calculations.

#### 3.4.2 Age

Incidence rates were highest in the youngest age categories in both primary care and EDs. In primary care, the yearly incidence rates by age category (< 6 months, < 1, < 2, and < 5 years) ranged from 7.2 to 330 (median = 202), 14.2 to 263 (median = 176), 19.0 to 263 (median = 109), and 0.8 to 212 (median = 35) per 1000 population, respectively. In the ED, the yearly incidence rates ranged from 74.8 to 144.0 (median = 112), 20.7 to 66.2 (median = 52), 26 to 75.7 (median = 44), and 7.5 to 40.4 (median = 28) per 1000 population by age category as above.

#### 3.4.3 Case Definition for RSV

In EDs, seven studies used bronchiolitis (ICD‐9 and ICD‐10 codes) as the case definition with non–laboratory‐confirmed RSV diagnosis. The overall yearly incidence rates reported by these studies were lower compared to studies conducted in EDs with laboratory‐confirmed RSV cases (Table 1). For example, in children aged < 1 year, the reported incidence rates ranged from 20.7 to 53.9 (median = 42) per 1000 population per year for non–laboratory‐confirmed (bronchiolitis) studies versus 34.4 to 144.0 (median = 71) for laboratory‐confirmed studies. Only three studies used bronchiolitis (non–laboratory confirmed) in primary care as the case definition, which was too little to evaluate the impact on the population‐based incidence rates.

#### 3.4.4 Country Income Level

In primary care, the RSV incidence rates were lower in lower‐middle‐income countries (LMICs) and upper‐middle‐income countries (UMICs) compared to high‐income countries (HICs), although only 4 out of 19 studies were conducted in LMICs/UMICs. In the < 1 year age category, the lowest reported yearly incidence rates (*n* = 3/16) were all from LMICs and UMICs, ranging from 7.2 to 79.8 (median = 14) per 1000 population compared to 89.9–330 (median = 214) per 1000 population in HICs. The same pattern was also observed in the age categories < 2 years and < 5 years. In the ED, only 1 study was conducted in a UMIC so the comparison between income levels could not be made.

## 4 Discussion

To our knowledge, this is the first systematic literature review that has provided a comprehensive overview of available literature on population‐based incidence rates of RSV infections in children < 5 years in primary care and EDs. The quality of the included studies to calculate incidence rates in a valid and unbiased manner was appraised as moderate. The reported incidence rates were higher in primary care compared to EDs. This was reflected in yearly RSV incidence rates that ranged from 0.8 to 330 (median = 109) per 1000 population in primary care, while in EDs, rates ranged from 7.5 to 144.0 (median = 48) per 1000 population. The highest RSV incidence rates were reported in infants under 1 year of age. Additionally, the case definition used for RSV and country income levels also seemed to impact RSV incidence rates. The reported incidence rates were higher in HICs compared to UMICs/LMICs, and the incidence rates in studies using laboratory‐confirmed RSV cases were also higher compared to studies using bronchiolitis ICD‐codes (non–laboratory confirmed). However, significant heterogeneity between study methodology and key characteristics was observed, making it challenging to draw definitive conclusions whether variability in reported incidence rates is due to disparities in care setting, age, case definition for RSV, and country income level.

Previous studies have focused on the hospital and community disease burden of RSV. Based on the expectations of the disease burden pyramid [6], a systematic analyses showed that global ALRI hospital admissions were estimated at 5.3 (4.2–6.8) per 1000 children [2], which is lower compared to our primary care and ED estimates. Contrary to our expectations, estimated RSV incidence rates in the community (48.8; 37.4–65.9, per 1000 children) [2] were similar compared to our ED estimates (48 per 1000) and lower compared to our primary care estimates (109 per 1000). However, the reported RSV estimates in the community might be underestimated, as RSV infections in the community are not tested and registered in a standard manner and not all infected children have a symptomatic course of disease [4]. Overall, we can conclude that RSV poses a substantial burden in young children in primary care and EDs.

It is known that RSV cases and especially deaths caused by RSV are significant in LMICs [2], but contrary to expectations, the incidence rates in LMICs and UMICs in this review were lower compared to HICs, although only four studies from these countries are included. The incidence rates in primary care might be lower in LMICs/UMICs due to limited access to healthcare facilities and different healthcare seeking behavior [46]. More information on the RSV burden in primary care and ED settings in LMICs and UMICs is therefore needed to fully capture the global RSV burden.

This is the first systematic review synthesizing RSV incidence rates in primary care settings and EDs. The results of this review can be used for future baseline RSV incidence rates, necessary to evaluate the effectiveness of the implementation of future preventative strategies. The most important limitation that should be considered while interpreting the findings is the high heterogeneity between studies related to contextual and methodological factors including setting, age, case definition for sampling and defining RSV, and country income level, but also world region, study method, national versus subnational, and seasonal versus year‐round data collection. There were insufficient studies with comparable contextual and methodological factors to perform (sensitivity) meta‐analyses for calculating pooled estimates. As a result, we only present medians and ranges. Consequently, it is impossible to draw conclusions about whether variations in incidence rates are attributable to, for example, disparities in RSV severity across years, or differences in healthcare settings and healthcare seeking behavior between countries. Additionally, bronchiolitis was used as proxy for RSV in this review, but it is known that using bronchiolitis ICD‐codes in hospital settings may lead to an overestimation of the burden of RSV [47]. Nevertheless, the use of bronchiolitis ICD‐codes in our review did not lead to higher RSV incidence rates, possibly instead to an underestimation compared to studies using surveillance data. Lastly, although ILI as case definition is not optimal for capturing RSV [48], no clear differences in RSV incidence rates were observed when ILI or ARI was used as case definition for sampling.

The quality of the included studies to calculate incidence rates of RSV in our targeted population was rated as moderate. This means that the study populations are not always completely representative for the target population, that is, studies reported incidence rates only for at‐risk children which might lead to an overestimation of the incidence rates, while others have excluded at‐risk patients from their study population. Furthermore, not all studies presented a 95% CI, making it difficult to determine the accuracy of the estimates. In addition, not all included studies described and reported all details of the study design, for example, the time period studied (year‐round or seasonal only). Another limitation is the generalizability of the results to all world regions as studies were mostly performed in HICs in North America and Europe. Lastly, approximately half of all studies relied on surveillance data—testing a random sample of cases for RSV—to estimate population‐based incidence rates in primary care, as testing for RSV in primary care is not part of routine standard care in most countries. This might result in an underestimation of the population‐based incidence rates.

Several studies suggest that RSV infections are frequently managed in outpatient settings [29, 44]. The burden of RSV is likely even higher than reported in this review in outpatient settings because viral detection tests are not standardly used and the diagnosis is mostly based on clinical findings. A high RSV disease burden in primary care leads to high pressure and demand on healthcare workers in the winter season. This leads to challenges and difficulties faced by healthcare facilities and providers in meeting the increased needs of patients seeking care for RSV‐related illness.

Harmonization of case definitions for RSV is crucial to guide public health interventions, allocate resources, and support policymaking aimed at reducing the burden of RSV infections. It is important to emphasize the need for a uniform RSV case definition in the interpretation of disease burden estimates, especially when based on surveillance data. The heterogeneity observed in this study highlights the challenges of comparing and generalizing the findings across different studies and settings. When estimating the burden of RSV, researchers should consider the potential impact of methodological and contextual differences between studies on the resulting disease burden estimates [32, 49]. Implementing a standardized case definition for RSV infections in young children would facilitate accurate and consistent estimation of disease burden, enabling better comparability and monitoring of trends over time.

Finally, it is important to emphasize that all incidence rates reported in this review were collected before the COVID‐19 pandemic. It is widely recognized that RSV activity was initially disrupted during the COVID‐19 pandemic followed by heightened awareness to RSV as pandemic measures were relaxed [50]; future research is necessary to show whether the disease burden of RSV will return to pre‐COVID‐19 levels.

## 5 Conclusions

This study showed that a substantial number of children < 5 years attend primary care and EDs every year for RSV infections. To fully understand the disease burden of RSV infections in young children in primary care and EDs, it is crucial to conduct studies with comparable study designs, using a uniform case definition for RSV and uniform age groups. Moreover, there is a need for additional studies in LMICs and UMICs. Disease burden estimates are important to support informed decision‐making regarding the introduction of future targeted interventions, including monoclonal antibodies and (maternal) vaccines.

## Author Contributions

**Susanne Heemskerk:** conceptualization, data curation, formal analysis, methodology, visualization, writing–original draft, writing–review and editing. **Lotte van Heuvel:** conceptualization, data curation, formal analysis, methodology, visualization, writing–review and editing. **Tamana Asey:** conceptualization, writing–review and editing. **Mathieu Bangert:** conceptualization, writing–review and editing. **Rolf Kramer:** conceptualization, writing–review and editing. **John Paget:** conceptualization, funding acquisition, writing–review and editing. **Jojanneke van Summeren:** conceptualization, data curation, formal analysis, methodology, visualization, writing–review and editing.

## Conflicts of Interest

S.H., L.v.H., and T.A. declare no competing interests. J.v.S. and J.P. declare that Nivel has received unrestricted research grants from WHO, Sanofi, and the Foundation for Influenza Epidemiology outside the submitted work. J.v.S. and J.P. declare that Nivel received a grant from the Respiratory Syncytial Virus Consortium in Europe (RESCEU) project of the “Innovative Medicines Initiative 2 Joint Undertaking” Grant Agreement No. 116019 and a grant from the Preparing for RSV Immunisation and Surveillance in Europe (PROMISE) project of the “Innovative Medicines Initiative 2 Joint Undertaking” Grant Agreement No. 101034339. This Joint Undertaking gets support from the “European Union's Horizon 2020 research and innovation programme” and the “European Federation of Pharmaceutical Industries and Associations”. M.B. and R.K. are employees of Sanofi and may hold shares and/or stock options in the company.

### Peer Review

The peer review history for this article is available at https://www.webofscience.com/api/gateway/wos/peer‐review/10.1111/irv.13344.

## Supporting information


**Supporting Information S1** Supplemental Digital Content 1. Search strategy.
**Supplemental Digital Content 2.** Detailed interpretation of JBI Critical Appraisal Checklist.
**Supplemental Digital Content 3.** Critical appraisal of the included studies.

**Supporting Information S2** Supplemental Digital Content 4. Data extraction table.

## Data Availability

Data is available in the article supplementary material.

## References

[1] M. Naghavi , A. A. Abajobir , C. Abbafati , et al., “Global, Regional, and National Age‐Sex Specific Mortality for 264 Causes of Death, 1980–2016: A Systematic Analysis for the Global Burden of Disease Study 2016,” The Lancet. 390, no. 10100 (2017): 1151–1210. 10.1016/S0140-6736(17)32152-9 PMC5605883 28919116

[2] Y. Li , X. Wang , D. M. Blau , et al., “Global, Regional, and National Disease Burden Estimates of Acute Lower Respiratory Infections due to Respiratory Syncytial Virus in Children Younger Than 5 Years in 2019: A Systematic Analysis,” Lancet 399, no. 10340 (2022): 2047–2064. 35598608 10.1016/S0140-6736(22)00478-0 PMC7613574

[3] T. Shi , D. A. McAllister , K. L. O'Brien , et al., “Global, Regional, and National Disease Burden Estimates of Acute Lower Respiratory Infections due to Respiratory Syncytial Virus in Young Children in 2015: A Systematic Review and Modelling Study,” Lancet 390, no. 10098 (2017): 946–958. 28689664 10.1016/S0140-6736(17)30938-8 PMC5592248

[4] J. G. Wildenbeest , M. N. Billard , R. P. Zuurbier , et al., “The Burden of Respiratory Syncytial Virus in Healthy Term‐Born Infants in Europe: A Prospective Birth Cohort Study,” The Lancet Respiratory Medicine 11, no. 4 (2023): 341–353. 36372082 10.1016/S2213-2600(22)00414-3 PMC9764871

[5] H. Nair , V. R. Verma , E. Theodoratou , et al., “An Evaluation of the Emerging Interventions Against Respiratory Syncytial Virus (RSV)‐Associated Acute Lower Respiratory Infections in Children,” BMC Public Health 11 (2011): S30. 21501449 10.1186/1471-2458-11-S3-S30 PMC3231904

[6] Centers for Disease Control and Prevention , “Burden of Flu,” accessed July 19, 2023, https://www.cdc.gov/flu/about/burden/index.html.

[7] R. Barr , C. A. Green , C. J. Sande , and S. B. Drysdale , “Respiratory Syncytial Virus: Diagnosis, Prevention and Management,” Therapeutic Advances in Infectious Disease 6 (2019): 2049936119865798. 31384456 10.1177/2049936119865798 PMC6664627

[8] B. Resch , “Respiratory Syncytial Virus Infection in High‐Risk Infants–An Update on Palivizumab Prophylaxis,” The Open Microbiology Journal. 8 (2014): 71–77. 25132870 10.2174/1874285801408010071 PMC4133922

[9] L. Garegnani , L. Styrmisdóttir , P. Roson Rodriguez , C. M. Escobar Liquitay , I. Esteban , and J. V. Franco , “Palivizumab for Preventing Severe Respiratory Syncytial Virus (RSV) Infection in Children,” Cochrane Database of Systematic Reviews 11, no. 11 (2021): Cd013757. 34783356 10.1002/14651858.CD013757.pub2 PMC8594174

[10] PATH , “RSV Vaccine and mAb Snapshot,” accessed April 15, 2024, https://www.path.org/resources/rsv‐vaccine‐and‐mab‐snapshot/.

[11] L. L. Hammitt , R. Dagan , Y. Yuan , et al., “Nirsevimab for Prevention of RSV in Healthy Late‐Preterm and Term Infants,” The New England Journal of Medicine 386, no. 9 (2022): 837–846. 35235726 10.1056/NEJMoa2110275

[12] Z. Munn , S. Moola , K. Lisy , D. Riitano , and C. Tufanaru , “Methodological Guidance for Systematic Reviews of Observational Epidemiological Studies Reporting Prevalence and Cumulative Incidence Data,” International Journal of Evidence‐Based Healthcare 13, no. 3 (2015): 147–153. 26317388 10.1097/XEB.0000000000000054

[13] The World Bank , “World Bank Country and Lending Groups,” accessed May 19, 2023, https://datahelpdesk.worldbank.org/knowledgebase/articles/906519‐world‐bank‐country‐and‐lending‐groups.

[14] C. S. Ambrose , E. J. Anderson , E. A. Simões , et al., “Respiratory Syncytial Virus Disease in Preterm Infants in the U.S. Born at 32‐35 Weeks Gestation Not Receiving Immunoprophylaxis,” The Pediatric Infectious Disease Journal 33, no. 6 (2014): 576–582. 24622396 10.1097/INF.0000000000000219 PMC4025592

[15] E. Barbieri , S. Cavagnis , A. Scamarcia , et al., “Assessing the Burden of Bronchiolitis and Lower Respiratory Tract Infections in Children ≤24 Months of Age in Italy, 2012‐2019,” Frontiers in Pediatrics 11 (2023): 1143735. 37215598 10.3389/fped.2023.1143735 PMC10196108

[16] F. T. Bourgeois , C. Valim , A. J. McAdam , and K. D. Mandl , “Relative Impact of Influenza and Respiratory Syncytial Virus in Young Children,” Pediatrics 124, no. 6 (2009): e1072–e1080. 19933730 10.1542/peds.2008-3074 PMC3374864

[17] F. T. Bourgeois , C. Valim , J. C. Wei , A. J. McAdam , and K. D. Mandl , “Influenza and Other Respiratory Virus‐Related Emergency Department Visits Among Young Children,” Pediatrics 118, no. 1 (2006): e1–e8. 16818524 10.1542/peds.2005-2248

[18] J. Brunet , P. J. Gill , H. Imsirovic , et al., “Evaluation of Bronchiolitis‐Related Emergency Department Visits From 2004 to 2018: A Population‐Based Cohort Study,” JAMA Pediatrics 176, no. 7 (2022): 719–722. 35499845 10.1001/jamapediatrics.2022.0707 PMC9062766

[19] M. Bueno Campaña , C. Calvo Rey , M. C. Vázquez Alvarez , et al., “Viral Respiratory Tract Infections in the First Six Months of Life,” Anales de Pediatría (Barcelona, Spain) 69, no. 5 (2008): 400–405. 10.1157/13127993 PMC7105042 19128739

[20] D. Cromer , A. J. van Hoek , A. T. Newall , A. J. Pollard , and M. Jit , “Burden of Paediatric Respiratory Syncytial Virus Disease and Potential Effect of Different Immunisation Strategies: A Modelling and Cost‐Effectiveness Analysis for England,” The Lancet Public Health 2, no. 8 (2017): e367–e374. 28804787 10.1016/S2468-2667(17)30103-2 PMC5541134

[21] F. C. K. Dolk , P. T. de Boer , L. Nagy , et al., “Consultations for Influenza‐Like Illness in Primary Care in the Netherlands: A Regression Approach,” Value in Health 24, no. 1 (2021): 11–18. 33431142 10.1016/j.jval.2020.10.013

[22] J. Forster , G. Ihorst , C. H. Rieger , et al., “Prospective Population‐Based Study of Viral Lower Respiratory Tract Infections in Children Under 3 Years of Age (the PRI.DE Study),” European Journal of Pediatrics 163, no. 12 (2004): 709–716. 15372233 10.1007/s00431-004-1523-9

[23] C. B. Hall , G. A. Weinberg , M. K. Iwane , et al., “The Burden of Respiratory Syncytial Virus Infection in Young Children,” The New England Journal of Medicine 360, no. 6 (2009): 588–598. 19196675 10.1056/NEJMoa0804877 PMC4829966

[24] K. Hasegawa , Y. Tsugawa , D. F. Brown , J. M. Mansbach , and C. A. Camargo, Jr. , “Temporal Trends in Emergency Department Visits for Bronchiolitis in the United States, 2006 to 2010,” The Pediatric Infectious Disease Journal 33, no. 1 (2014): 11–18. 23934206 10.1097/INF.0b013e3182a5f324 PMC3984903

[25] T. Heikkinen , E. Ojala , and M. Waris , “Clinical and Socioeconomic Burden of Respiratory Syncytial Virus Infection in Children,” The Journal of Infectious Diseases 215, no. 1 (2017): 17–23. 27738052 10.1093/infdis/jiw475

[26] M. L. Jackson , L. Starita , E. Kiniry , et al., “Incidence of Medically Attended Acute Respiratory Illnesses due to Respiratory Viruses Across the Life Course During the 2018/19 Influenza Season,” Clinical Infectious Diseases 73, no. 5 (2021): 802–807. 33590002 10.1093/cid/ciab131 PMC7929037

[27] T. Kamigaki , P. P. Aldey , E. S. Mercado , et al., “Estimates of Influenza and Respiratory Syncytial Virus Incidences With Fraction Modeling Approach in Baguio City, the Philippines, 2012‐2014,” Influenza and Other Respiratory Viruses 11, no. 4 (2017): 311–318. 28371393 10.1111/irv.12453 PMC5485869

[28] B. J. Law , J. M. Langley , U. Allen , et al., “The Pediatric Investigators Collaborative Network on Infections in Canada Study of Predictors of Hospitalization for Respiratory Syncytial Virus Infection for Infants Born at 33 Through 35 Completed Weeks of Gestation,” The Pediatric Infectious Disease Journal 23, no. 9 (2004): 806–814. 15361717 10.1097/01.inf.0000137568.71589.bd

[29] J. Y. Lively , A. T. Curns , G. A. Weinberg , et al., “Respiratory Syncytial Virus‐Associated Outpatient Visits Among Children Younger Than 24 Months,” Journal of the Pediatric Infectious Diseases Society 8, no. 3 (2019): 284–286. 10.1093/jpids/piz011 30840770

[30] J. M. Mansbach , J. A. Emond , and C. A. Camargo, Jr. , “Bronchiolitis in US Emergency Departments 1992 to 2000: Epidemiology and Practice Variation,” Pediatric Emergency Care 21, no. 4 (2005): 242–247. 15824683 10.1097/01.pec.0000161469.19841.86

[31] J. M. Mansbach , A. J. Pelletier , and C. A. Camargo, Jr. , “US Outpatient Office Visits for Bronchiolitis, 1993‐2004,” Ambulatory Pediatrics 7, no. 4 (2007): 304–307. 17660102 10.1016/j.ambp.2007.03.006

[32] D. N. Marcone , L. O. Durand , E. Azziz‐Baumgartner , et al., “Incidence of Viral Respiratory Infections in a Prospective Cohort of Outpatient and Hospitalized Children Aged ≤5 Years and Its Associated Cost in Buenos Aires, Argentina,” BMC Infectious Diseases 15 (2015): 447. 26497393 10.1186/s12879-015-1213-4 PMC4619328

[33] H. C. Moore , N. de Klerk , P. Jacoby , P. Richmond , and D. Lehmann , “Can Linked Emergency Department Data Help Assess the Out‐of‐Hospital Burden of Acute Lower Respiratory Infections? A Population‐Based Cohort Study,” BMC Public Health 12 (2012): 703. 22928805 10.1186/1471-2458-12-703 PMC3519642

[34] C. Muñoz‐Quiles , M. López‐Lacort , I. Úbeda‐Sansano , et al., “Population‐Based Analysis of Bronchiolitis Epidemiology in Valencia, Spain,” The Pediatric Infectious Disease Journal 35, no. 3 (2016): 275–280. 26658376 10.1097/INF.0000000000000993

[35] E. A. Okiro , M. Ngama , A. Bett , and D. J. Nokes , “The Incidence and Clinical Burden of Respiratory Syncytial Virus Disease Identified Through Hospital Outpatient Presentations in Kenyan Children,” PLoS ONE 7, no. 12 (2012): e52520. 23300695 10.1371/journal.pone.0052520 PMC3530465

[36] W. J. Paget , C. Balderston , I. Casas , et al., “Assessing the Burden of Paediatric Influenza in Europe: The European Paediatric Influenza Analysis (EPIA) Project,” European Journal of Pediatrics 169, no. 8 (2010): 997–1008. 20229049 10.1007/s00431-010-1164-0 PMC2890072

[37] N. Prasad , A. A. Trenholme , Q. S. Huang , J. Duque , C. C. Grant , and E. C. Newbern , “Respiratory Virus‐Related Emergency Department Visits and Hospitalizations Among Infants in New Zealand,” The Pediatric Infectious Disease Journal 39, no. 8 (2020): e176–e182. 32675757 10.1097/INF.0000000000002681

[38] G. Rainisch , B. Adhikari , M. I. Meltzer , and G. Langley , “Estimating the Impact of Multiple Immunization Products on Medically‐Attended Respiratory Syncytial Virus (RSV) Infections in Infants,” Vaccine 38, no. 2 (2020): 251–257. 31740097 10.1016/j.vaccine.2019.10.023 PMC7029767

[39] A. I. Reyes Domínguez , S. Pavlovic Nesic , L. Urquía Martí , M. D. C. Pérez González , D. Reyes Suárez , and F. García‐Muñoz Rodrigo , “Effects of Public Health Measures During the SARS‐CoV‐2 Pandemic on the Winter Respiratory Syncytial Virus Epidemic: An Interrupted Time Series Analysis,” Paediatric and Perinatal Epidemiology 36, no. 3 (2022): 329–336. 34981845 10.1111/ppe.12829

[40] R. J. Rosychuk , T. P. Klassen , D. C. Voaklander , A. Senthilselvan , and B. H. Rowe , “Presentations of Infants to Emergency Departments in Alberta, Canada, for Bronchiolitis: A Large Population‐Based Study,” Pediatric Emergency Care 27, no. 3 (2011): 189–195. 21346678 10.1097/PEC.0b013e31820d650f

[41] E. Rowlinson , E. Dueger , T. Taylor , et al., “Incidence and Clinical Features of Respiratory Syncytial Virus Infections in a Population‐Based Surveillance Site in the Nile Delta Region,” The Journal of Infectious Diseases 208, no. Suppl 3 (2013): S189–S196. 24265478 10.1093/infdis/jit457

[42] M. Suh , N. Movva , X. Jiang , et al., “Respiratory Syncytial Virus Burden and Healthcare Utilization in United States Infants <1 Year of Age: Study of Nationally Representative Databases, 2011‐2019,” The Journal of Infectious Diseases 226, no. Suppl 2 (2022): S184–s194. 35968879 10.1093/infdis/jiac155 PMC9377028

[43] S. Tempia , J. Moyes , A. L. Cohen , et al., “The National Burden of Influenza‐Like Illness and Severe Respiratory Illness Overall and Associated With Nine Respiratory Viruses in South Africa, 2013‐2015,” Influenza and Other Respiratory Viruses 16, no. 3 (2022): 438–451. 35150059 10.1111/irv.12949 PMC8983907

[44] E. Thomas , J. M. Mattila , P. Lehtinen , T. Vuorinen , M. Waris , and T. Heikkinen , “Burden of Respiratory Syncytial Virus Infection During the First Year of Life,” The Journal of Infectious Diseases 223, no. 5 (2021): 811–817. 33350450 10.1093/infdis/jiaa754

[45] To T , E. Terebessy , J. Zhu , et al., “Did Emergency Department Visits in Infants and Young Children Increase in the Last Decade?: An Ontario, Canada Study,” Pediatric Emergency Care 38, no. 4 (2022): e1173–e1178. 34570077 10.1097/PEC.0000000000002535

[46] Our World in Data , “Healthcare Access and Quality by Level of Healthcare Spending,” accessed August 1, 2023, https://ourworldindata.org/grapher/HAQ‐by‐level‐of‐healthcare‐spending‐endpoints.

[47] T. A. Florin , A. C. Plint , and J. J. Zorc , “Viral Bronchiolitis,” Lancet 389, no. 10065 (2017): 211–224. 27549684 10.1016/S0140-6736(16)30951-5 PMC6765220

[48] World Health Organization , “RSV Surveillance Case Definitions,” accessed April 22, 2024, https://www.who.int/teams/global‐influenza‐programme/global‐respiratory‐syncytial‐virus‐surveillance/case‐definitions.

[49] K. M. Roguski , M. A. Rolfes , J. S. Reich , et al., “Variability in Published Rates of Influenza‐Associated Hospitalizations: A Systematic Review, 2007‐2018,” Journal of Global Health 10, no. 2 (2020): 020430. 33274066 10.7189/jogh.10.020430 PMC7699004

[50] J. van Summeren , A. Meijer , G. Aspelund , et al., “Low Levels of Respiratory Syncytial Virus Activity in Europe During the 2020/21 Season: What Can We Expect in the Coming Summer and Autumn/Winter?” Euro Surveillance 26, no. 29 (2021): 2100639. 34296672 10.2807/1560-7917.ES.2021.26.29.2100639 PMC8299745

