# Supplementary material for: Disease Burden of RSV Infections and Bronchiolitis in Young Children (< 5 Years) in Primary Care and Emergency Departments: A Systematic Literature Review
Source: Influenza Other Respir Viruses. 2024 Aug 4;18(8):e13344. doi: 10.1111/irv.13344 (PMC11298312; doi:10.1111/irv.13344)
Supplement: Supplementary file 1 — Supporting Information S1 Supplemental Digital Content 1. Search strategy. Supplemental Digital Content 2. Detailed interpretation of JBI Critical Appraisal Checklist. Supplemental Digital Content 3. Critical appraisal of the included studies. [file IRV-18-e13344-s001.docx]

**Supplemental Digital Content 1.** Search strategy

| **#1 Pathogen and disease** |
| --- |
| Pubmed: (RSV OR Respiratory Syncytial Virus OR Respiratory Syncytial Virus Infections OR Bronchiolitis OR VRS NOT “Verbal Rating Scale” NOT “variable returns to scale”) |
| Embase: (RSV OR Respiratory Syncytial Virus OR Respiratory Syncytial Virus Infection* OR Bronchiolitis OR VRS NOT ‘Verbal Rating Scale’ NOT ‘variable returns to scale’) |
| **#2 Setting and healthcare provider** |
| Pubmed: (Outpatient* OR General Practitioner OR GP OR Family Practitioner OR primary care OR ambulatory care OR pediatric* OR Emergency Department OR Emergency Room OR ED OR ENT) |
| Embase: (Outpatient* OR General Practitioner OR GP OR Family Practitioner OR primary care OR ambulatory care OR p?diatric* OR Emergency Department OR Emergency Room OR ED OR ENT) |

**Supplemental Digital Content 2: Detailed interpretation of JBI Critical Appraisal Checklist**

Answers: Yes, No, or Unclear

1. **Was the sample frame appropriate to address the target population?**

Score yes if all answers are yes:

- Boys and girls included

- Age range <5 years

- Inclusion of children in primary care and emergency departments (not hospitalized)

- Both at risk/not at risk patients are reported (score no if only at risk patients are reported)

- Inclusion of RSV cases (score no if bronchiolitis cases are reported)

1. **Were study participants recruited in an appropriate way?**

Score yes if:

- Random sampling methods

1. **Was the sample size adequate?**

→ not applicable

1. **Were the study subjects and setting described in detail?**

Score yes if all answers are yes:

- Description of age characteristics

- Description of setting (ED, OPD, GP office etc.)

- Description of country/region

- Inclusion/Exclusion criteria described

1. **Was data analysis conducted with sufficient coverage of the identified sample?**

→ not applicable

1. **Were valid methods used for the identification of the condition?**

Score yes if:

- Data source described (surveillance data, registry data, laboratory testing, etc.)

- RSV case definition based on official case definition (ILI, (S)ARI, bronchiolitis, etc.)

- In case of lab confirmed RSV: laboratory testing methods described (culture, nucleic test, antigen test, antibody test, etc.)

1. **Was the condition measured in a standard, reliable way for all participants?**

Score yes if:

- RSV testing is similar for all patients

- One physician made all clinical RSV diagnosis or;

- RSV diagnosis by well-trained or educated physicians (bronchiolitis)

1. **Was there appropriate statistical analysis?**

Score yes if:

- Incidence rates of RSV/bronchiolitis reported

- Statistical analysis described (regression analysis, modelling, etc.)

- Numerator and denominator are reported

- 95% CI mentioned

1. **Was the response rate adequate, and if not, was the low response rate managed appropriately?**

→ not applicable

**Supplemental Digital Content 3: Critical appraisal of the included studies**

| **Studies** | 1. Was the sample frame appropriate to address the target population? | 2. Were study participants recruited in an appropriate way? | 3. Were the study subjects and the setting described in detail? | 4. Were valid methods used for the identification of the condition? | 5. Was the condition measured in a standard, reliable way for all participants? | 6. Was there appropriate statistical analysis? |
| --- | --- | --- | --- | --- | --- | --- |
| Ambrose (2014)^14^ | No | Yes | Yes | Yes | Yes | Yes |
| Barbieri (2023)^15^ | No | Yes | Yes | Yes | Yes | Yes |
| Bourgeois (2009)^16^ | Yes | No | Yes | Yes | Yes | Yes |
| Bourgeois (2006)^17^ | Yes | Yes | Yes | Yes | Yes | Yes |
| Brunet (2022)^18^ | No | Yes | Yes | Yes | Yes | Yes |
| Bueno Campaña (2008)^19^ | Yes | Unclear | Yes | Yes | Yes | No |
| Cromer (2017)^20^ | Yes | Unclear | Yes | Unclear | Yes | Yes |
| Dolk (2021)^21^ | Yes | Yes | Yes | Yes | Yes | No |
| Forster (2004)^22^ | Yes | Yes | Yes | Yes | Yes | Yes |
| Hall (2009)^23^ | Yes | Yes | Yes | Yes | Yes | Yes |
| Hasegawa (2014)^24^ | No | Yes | Yes | Yes | Yes | No |
| Heikkinen (2017)^25^ | Yes | Yes | Yes | Yes | Yes | Yes |
| Jackson (2021)^26^ | Yes | Unclear | Yes | Yes | Yes | No |
| Kamigaki (2017)^27^ | Yes | Yes | Yes | Yes | Yes | Yes |
| Law (2004)^28^ | No | Unclear | Yes | Yes | Yes | No |
| Lively (2019)^29^ | Yes | Yes | Yes | Yes | Yes | Yes |
| Mansbach (2005)^30^ | No | Yes | Yes | Yes | Yes | Yes |
| Mansbach (2007)^31^ | No | Yes | Yes | Yes | Yes | Yes |
| Marcone (2015)^32^ | Yes | No | Yes | Yes | Yes | Yes |
| Moore (2012)^33^ | No | Unclear | Yes | Yes | Yes | No |
| Muñoz- Quiles (2016)^34^ | No | Yes | Yes | Yes | Yes | No |
| Okiro (2012)^35^ | Yes | Yes | Yes | Yes | Yes | Yes |
| Paget (2010)^36^ | Yes | Yes | Yes | Yes | Yes | No |
| Prasad (2020)^37^ | Yes | Yes | Yes | Yes | Yes | Yes |
| Rainisch (2020)^38^ | Yes | Unclear | Yes | Unclear | Yes | Yes |
| Reyes Dominguez (2022)^39^ | Yes | Unclear | Yes | Yes | Yes | No |
| Rosychuk (2011)^40^ | No | Unclear | Yes | Yes | Yes | No |
| Rowlinson (2013)^41^ | Yes | Yes | Yes | Yes | Yes | Yes |
| Suh (2022)^42^ | Yes | Unclear | Yes | Yes | Yes | Yes |
| Tempia (2022)^43^ | Yes | Unclear | Yes | Yes | Yes | Yes |
| Thomas (2021)^44^ | Yes | Unclear | Yes | Yes | Yes | Yes |
| To (2022)^45^ | No | Unclear | Yes | Yes | Yes | No |

‘‘Yes’’ means no risk of bias, ‘‘no’’ means risk of bias, and ‘‘unclear’’ means the study authors provided either no information or incomplete information.
